# RANKL and RANK in extracellular vesicles: surprising new players in bone remodeling

**DOI:** 10.20517/evcna.2020.02

**Published:** 2021-03-30

**Authors:** L. Shannon Holliday, Shivani S. Patel, Wellington J., Jr Rody

**Affiliations:** ^1^Department of Orthodontics, University of Florida College of Dentistry, Gainesville, FL 32610, USA.; ^2^Department of Orthodontics and Pediatric Dentistry, Stony Brook School of Dental Medicine, Stony Brook, NY 11794, USA.; ^3^Department of Anatomy & Cell Biology, University of Florida College of Medicine, Gainesville, FL 32610, USA.

**Keywords:** Cell signaling, osteoclast, osteoblast, osteocyte, exosome, microvesicle, coupling factor, apoptotic vesicle

## Abstract

Receptor activator of nuclear factor kappa B-ligand (RANKL), its receptor RANK, and osteoprotegerin which binds RANKL and acts as a soluble decoy receptor, are essential controllers of bone remodeling. They also play important roles in establishing immune tolerance and in the development of the lymphatic system and mammary glands. In bone, RANKL stimulates osteoclast formation by binding RANK on osteoclast precursors and osteoclasts. This is required for bone resorption. Recently, RANKL and RANK have been shown to be functional components of extracellular vesicles (EVs). Data linking RANKL and RANK in EVs to biological regulatory roles are reviewed, and crucial unanswered questions are examined. RANKL and RANK are transmembrane proteins and their presence in EVs allows them to act at a distance from their cell of origin. Because RANKL-bearing osteocytes and osteoblasts are often spatially distant from RANK-containing osteoclasts in vivo, this may be crucial for the stimulation of osteoclast formation and bone resorption. RANK in EVs from osteoclasts has the capacity to stimulate a RANKL reverse signaling pathway in osteoblasts that promotes bone formation. This serves to couple bone resorption with bone formation and has inspired novel bifunctional therapeutic agents. RANKL- and RANK- containing EVs in serum may serve as biomarkers for bone and immune pathologies. In summary, EVs containing RANKL and RANK have been identified as intercellular regulators in bone biology. They add complexity to the central signaling network responsible for maintaining bone. RANKL- and RANK-containing EVs are attractive as drug targets and as biomarkers.

## INTRODUCTION

Receptor activator of nuclear factor kappa B-ligand (RANKL), its receptor RANK, and the RANKL-binding decoy receptor osteoprotegerin are members of the tumor necrosis factor (TNF) and TNF receptor superfamilies^[1]^. In the late 1990s, these proteins were identified as essential for regulating the formation of osteoclasts and thus for bone remodeling^[2,3]^. Mice in which RANKL was knocked out had few or no osteoclasts, the hematopoietic cells specialized to resorb bone, and were severely osteopetrotic^[4]^. In addition, these mice had immune cell abnormalities and lacked certain lymph nodes. Female RANKL knockout mice died before maturity as a consequence of defective breast development^[5]^. Mice in which osteoprotegerin was knocked out were osteoporotic^[2]^. This led to the hypothesis that bone resorption was a function of the ratio of RANKL to osteoprotegerin. 

These regulatory molecules were identified due to efforts to find the “osteoclast differentiation factor”. Indirect evidence had suggested that there might be a specific regulatory molecule responsible for directing hematopoietic stem cells to differentiate into osteoclasts. Since bone diseases, in particular osteoporosis and metastatic bone cancer, are major clinical problems that are associated with excess bone resorption by osteoclasts, it was reasoned that if such a specific regulatory molecule existed, it would be an extremely attractive target for drug development^[6,7]^. Groups from Amgen and Snow Brand Milk Products concurrently identified RANKL (called “osteoprotegerin-ligand” by the Amgen group and “osteoclast differentiation factor” by the group from Snow Brand) as the osteoclast differentiation factor^[2,3]^. The nomenclature was later resolved to RANKL. Osteoprotegerin, which binds RANKL and blocks its interaction with RANK, and thus osteoclast formation, has not been developed directly into a therapeutic agent because it is promiscuous, binding other TNF superfamily members, and has a short half-life in circulation^[6]^. Amgen developed a humanized monoclonal antibody directed against RANKL (denosumab), which has become a major therapeutic for the treatment of osteoporosis (Prolia*^ ®^*) and bone cancer (Xgeva*^®^*)^[7-9]^. Although RANKL, RANK, and osteoprotegerin have roles in the immune system, the use of denosumab as a therapeutic has had relatively few off target effects.

Extracellular vesicles (EVs) include exosomes and microvesicle (also known as ectosomes)^[10,11]^. Exosomes are released from cells as cytosolic multivesicular bodies fuse with the plasma membrane. The multivesicular bodies are formed by inward budding of the bounding membrane of cytosolic vesicles. Microvesicles are formed by direct budding from the plasma membrane. Evidence suggests that there is considerable overlap in the composition and size range of exosomes and microvesicles. For this reason, the International Society for Extracellular Vesicles recommends using the term EV to describe these vesicles unless there is a clear evidence that the EVs are exosomes or microvesicles^[12]^. In this article we will use the term EV, though various nomenclatures were used in the original reports. A third type of EV is apoptotic vesicles that form during apoptosis, but these are larger and are usually separated from exosomes and microvesicles during standard isolations. During the past decade, many studies have supported the hypothesis that EVs serve as intercellular regulators^[13]^.

**Figure 1:**
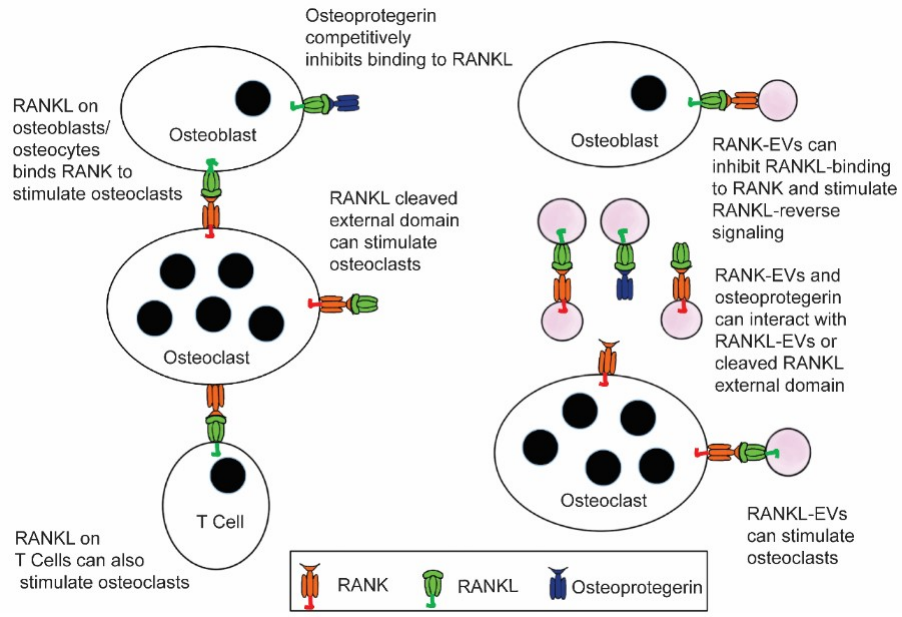
Extracellular vesicles containing RANKL or RANK add increased complexity to the RANKL/RANK/osteoprotegerin signaling network that is at the core of bone biology. Until recently, RANKL stimulation of RANK to stimulate osteoclast formation and bone resorption by osteoclasts, and the ability of osteoprotegerin to bind RANKL and competitively inhibit this signaling, were considered the primary features of this network. Now data suggest that RANKL EVs can stimulate osteoclast formation and bone resorption through RANK stimulation, and RANK-EVs bind to RANKL on osteoblasts to stimulate RANKL reverse signaling and bone formation. The latter serves to couple bone resorption and bone formation. It is also possible that RANK-EVs can bind RANKL or RANKL-EVs and competitively inhibit their stimulation of RANK on osteoclasts. RANKL-EVs could serve as competitive inhibitors of RANK-EV’s stimulation of RANKL reverse signaling.

The discovery of RANKL in EVs in 2015^[14]^, and RANK in EVs in 2016^[15]^, together with other recent advances in the understanding of bone biology, particularly the finding that most RANKL that stimulates bone resorption is from osteocytes^[16-20]^, has opened up new lines of study [Figure 1]. Incorporation of these transmembrane proteins into EVs allows each to be, as needed, either a receptor or a ligand, serving as an elegant and parsimonious evolutionary solution for regulating the coupling of bone resorption and bone formation. As this work develops, it promises deeper understanding of how bone is remodeled, and new therapeutic approaches to treat bone disease. However, numerous unanswered questions are open in this field of research.

## RANKL-EVS

### Osteoblast EVs: long range stimulation of osteoclastic bone resorption

RANKL is expressed by osteoblasts, osteocytes, T cells, B cells, and various other tissues during development^[4,19,20]^. It is also found in the brain in the lateral septal nucleus^[21]^, and astrocytes and microglia contain RANK and respond to RANKL^[22,23]^. RANKL is a transmembrane protein that can also act as a receptor^[24]^. RANKL exists in cells as a homotrimer. The binding site for RANK (also a homotrimer) on RANKL is formed by elements of two adjacent RANKL molecules^[25]^. Until the discovery of RANKL in EVs, two forms of RANKL were known in biology, the intact membrane bound form, and a soluble form cleaved from the plasma membrane by exoproteases/sheddases including TNF-α converting enzyme, several a disintegrin and metalloproteinases, and matrix metalloproteinases^[26-28]^. A recent study showed that in mice where RANKL was altered to prevent the action of exoproteases, normal bone physiology was not perturbed, although the mice were more susceptible to cancer invasion^[29]^.

Data suggesting regulatory RANKL in EVs was first described in an *in vitro *system^[14]^. EVs isolated from a stromal/osteoblastic cell line (UAMS-32P) contained RANKL and were able to stimulate differentiation of RAW 264.7 cells into osteoclast-like cells. Immunogold transmission electron microscopy indicated that the EVs containing RANKL were large (200 nm or more), suggesting that they may be microvesicles. While this report utilized cell lines, it provided the first suggestion of functional RANKL in EVs.

These data were confirmed and extended to show that EVs released by primary osteoblasts (isolated from mouse calvaria) produced RANKL-containing EVs that stimulated primary mouse monocytes to differentiate into osteoclasts^[30]^. These EVs were also large, 100-200 nm in diameter, suggesting that they may be microvesicles. In addition, these RANKL-containing EVs triggered osteoclast formation *in vivo* when injected into transgenic mice in which RANKL was knocked out. By using osteoblast EVs loaded with the fluorophore chloromethyl fluorescein diacetate, it was shown that the osteoblast-derived EVs could fuse with target cells and deliver the luminal contents to the cytosol. It was then demonstrated that RANKL-containing EVs could be used to deliver anti-osteoclastic drugs to osteoclasts *in vivo*. Overactive osteoclasts were stimulated by treatment of mice with retinoic acid. These mice were then injected with RANKL-containing EVs from osteoblasts in which the osteoclast inhibitors, zoledronic acid (which blocks prenylation pathways) or dasatinib (which blocks tyrosine kinases including c-src) had been incorporated. The therapeutic efficacy of the EVs was demonstrated by the reduction of the overstimulation of osteoclasts, caused by retinoic acid, measured by both a serum marker of bone resorption and counts of osteoclasts and apoptotic osteoclasts in bone sections^[30]^. In a larger sense, these results suggest that in addition to stimulating RANK on the surface of the osteoclasts, the RANKL-EVs fused with and delivered the contents of the lumen of the EVs (osteoclast inhibiting pharmaceuticals) to the target cells. While in this case, the lumens contained zoledronic acid or dastinib, it suggests that under physiologic conditions, regulatory agents found in the EV lumen (microRNAs for example) are targeted to the cytosol of osteoclasts. Characterization of the composition of RANKL-EVs shed by osteoblasts will be required to identify other regulatory signals that may be present.

Recently, a zebra-fish model using fluorescently-tagged proteins for direct visualization of EVs was used to demonstrate that osteoblasts release RANKL-containing EVs that stimulate osteoclast formation *in vitro*, and also deliver luminal contents to osteoclasts *in vivo*^[31]^. These data confirmed the results of the studies in mammalian cells and suggest that the involvement of RANKL-containing EVs in bone signaling has ancient evolutionary origins.

Studies have suggested that EVs from osteoblasts and osteocytes are involved in communication with each other, with mesenchymal stem cells, and with myoblasts^[32]^. Osteoblast EVs contain various microRNAs, but the receptor-ligand binding interactions that dock the EVs have not been identified. It is also not clear whether microRNAs or other type of RNAs carried in EVs are acting in these regulatory pathways.

### RANKL in osteocyte EVs

Osteocytes have been shown to release regulatory EVs^[33-38]^ and RANKL was shown to be released by in EVs from osteocytes in response to mechanical force^[34]^. However, it has not yet been shown that RANKL-containing EVs shed by osteocytes stimulate biologically relevant osteoclast formation. It has emerged that most (90%) of the RANKL stimulation of osteoclasts *in vivo* in mammals is by RANKL derived from osteocytes^[17]^. These cells are a final differentiation step in the osteoblast lineage and are the most abundant cells in bone, inhabiting lacunae that weave through the bone. It is not clear how RANKL on the surface of osteocytes would be accessible to osteoclasts and their precursors. One possibility is that soluble RANKL cleaved by proteases might serve to stimulate distant osteoclasts, but recent data indicating soluble RANKL is not required for normal bone physiology suggest that this is not the case^[29]^. It is attractive to hypothesize that RANKL-containing EVs released from osteocytes have the ability to stimulate spatially distant osteoclasts.

## RANK-EVS

### Osteoclast EVs: RANKL reverse signaling

RANK was first identified in EVs *in vitro* in efforts to identify the basis of differential regulatory activities of EVs released by pre-osteoclasts compared with EVs released from mature osteoclasts^[15]^. Several differentially expressed proteins were detected, and mass spectrometry identified RANK to be relatively abundant in EVs from osteoclasts, but not in EVs from precursors. This result was confirmed by immunoblots and by immunogold labeling of EVs from osteoclasts visualized in negative stained transmission electron microscopy. The electron microscopy indicated that RANK in EVs was concentrated in a small subset of the total EVs shed by osteoclasts. The size of the EVs in which RANK was detected was about 50-nm in diameter, which is consistent with the RANK-containing EVs being exosomes. Data were also presented that suggested that the RANK-containing EVs inhibited osteoclast formation in calcitriol-stimulated mouse marrow cultures by a paracrine mechanism. It was suggested that RANK-EVs might act as competitive inhibitors of the RANKL-RANK interaction in the manner of osteoprotegerin^[15,39]^.

The publication showing RANK in EVs appeared concurrently with two other article describing regulatory EVs released by osteoclasts^[40,41]^. Both of these reports found that EVs from osteoclasts delivered microRNA-214-3p to osteoblasts and consequent reduction of target mRNA translation inhibited osteoblast formation and function. One group reported that targeting of the EVs from osteoclasts to osteoblasts was achieved by interaction between semaphorin 4D on the osteoclast EVs with plexinB1 on the osteoblast^[40]^. The semaphorin 4D-containing EVs then fused with the osteoblast to release luminal microRNA 214-3p into the cytosol where it inhibited translation of the mRNA coding for activating transcription factor 4. This resulted in reduced osteoblast formation and bone formation^[40]^. The second study found that osteoclast EVs bound to osteoblasts through ephrinA2 binding to ephrin receptor on the osteoblasts. They also reported that osteoblasts were inhibited due to the introduction of microRNA 214-3p into the osteoblast cytosol. Both of these studies found EVs from osteoclasts to be inhibitors of osteoblasts^[40,41]^. These studies were not consistent with EVs from osteoclasts serving as “coupling factors” that signal to balance bone formation with bone resorption.

The initial finding of RANK in EVs was subsequently confirmed by several groups^[42-44]^. Ikebuchi* et al.*^[24]^ greatly expanded the understanding of RANK-containing EVs in an article in *Nature. *They showed that when RANK-containing EVs bind RANKL on osteoblasts, a RANKL reverse signaling pathway is stimulated that triggers pre-osteoblastic cells, which do not form bone, to differentiate and form bone. This reverse signaling pathway acted through phosphatidylinositol 3-kinase, mammalian target of rapamycin and runt-related transcription factor 2. They hypothesized that the RANK-containing EVs would act as “coupling factors” that could promote bone formation to replace bone resorbed by osteoclasts^[45,46]^. They demonstrated *in vivo* that RANK-EVs promoted bone formation in the calvarial critical size bone defect model. In an exciting addition, they found that certain cross-linked anti-RANKL antibodies mimicked RANK-containing EVs to stimulate RANKL reverse signaling. These antibodies were tested in an *in vivo* rodent model of postmenopausal osteoporosis. They blocked the ability of RANKL to stimulate RANK, like osteoprotegerin, and thus blocked bone resorption. Unlike osteoprotegerin, they also stimulated RANKL reverse signaling and bone formation. This provided a proof-of-concept for a new type of bifunctional therapeutic agent that may have the potential to bring about transformative changes to the way osteoporosis and other bone diseases are treated.

### Regulation of RANK-EVs release by osteoclasts

It is attractive to hypothesize that the shedding of RANK-containing EVs is regulated. One line of evidence supporting this idea is the finding that osteoclasts resorbing bone released 5-times more RANK than the same cells resorbing dentin, the mineralized tissue that makes up the bulk of a tooth^[47]^. The differential release of RANK in EVs was accompanied by differential release of numerous other components as determined by quantitative high-resolution liquid chromatograph-mass spectrometry. The difference in RANK-containing EV shedding observed in this study could have been driven by matrix-related factors. This is biologically plausible in that tooth resorption is not coupled to bone formation. Moreover, if coupled bone formation occurred associated with tooth resorption, it could lead to fusion of the alveolar bone to the tooth structure, a pathological process named ankylosis. It is of interest to identify the factors in bone that trigger RANK-containing EV release, or perhaps the factors that suppress release when the cells are resorbing dentin. Although the two mineralized tissue share many components, previous studies show that there are compositional differences^[48-50]^.

**Figure 2:**
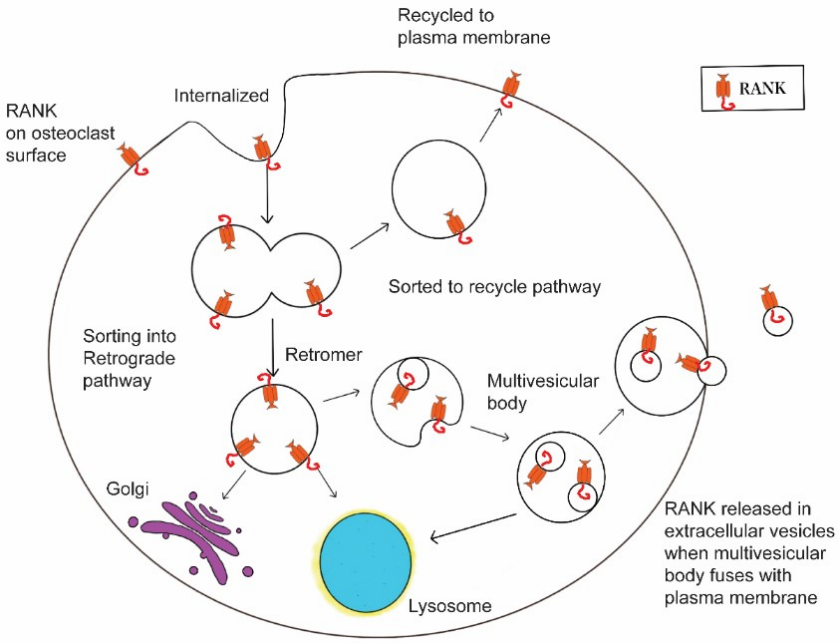
Regulation of RANK-containing EV production. We hypothesize that when RANK is internalized, an element of the normal receptor life cycle, it can either be recycled back to the plasma membrane, where it would be available for continued stimulation by RANKL. This would favor bone resorption. Alternatively, it could be sorted into a retrograde pathway that could lead to the Golgi for reuse of RANK, to the lysosome for degradation, or to multivesicular bodies, some of which would fuse with the plasma membrane and release RANK-EVs. All the retrograde pathways would remove RANK from the plasma membrane surface, and thus reduce stimulation by RANKL. In addition, shedding of RANK-EVs would also stimulate the RANKL reverse pathway and bone formation. If this idea is correct, shifts in the sorting of RANK could have profound effects on both bone resorption and bone formation, and understanding the underlying mechanisms may identify new strategies for treating bone disease.

What about other factors? For example, do specific cytokines known to regulate osteoclast formation and activity also activate, or suppress RANK-EV release? Might mechanical stress, which favors bone formation *in vivo*^[51,52]^, stimulate RANK-EV shedding in the same way as it does with osteocytes? At a more fundamental level, what mechanisms are involved in packaging RANK into EVs? For example, are receptors incorporated into EVs, in this case exosomes, as a pathway that is part of the general receptor recycling process [Figure 2]? RANK recycling after internalization from the plasma membrane has been shown to be controlled by the retromer complex^[53]^. Reduction in the vps35 component of the retromer leads to more RANK on the osteoclast surface, and increased bone resorption that is uncoupled with bone formation in mice leading to osteoporosis^[53]^. We suggest that RANK recycling may simultaneously regulate the number of RANK molecules on the osteoclast cell surface that are available for stimulation by RANKL, and the number of RANK molecules loaded into exosomes. Regulation of the recycling process could serve as an osteoclast-centered rheostat regulating the amount of osteoclastic bone resorption and the relative amount of bone formation coupled to the bone resorption.

### RANK-EVs in apoptotic bodies from osteoclasts

A number of recent studies have suggested a change in paradigm; apoptotic bodies/vesicles may serve not only as easily handled materials for cells tasked with cleaning the dead cells remains, but also as regulatory signals^[13]^. In particular, apoptotic vesicles have been shown to produce pro-inflammatory signals. Apoptosis of osteoclasts typically precedes the recruitment and differentiation of osteoblasts in a bone remodeling unit^[45]^. Ma *et al.*^[54] ^explored whether apoptotic bodies might contain RANK and stimulate reverse RANKL signaling in the same way and RANK-containing EVs. They found that when osteoclasts were grown in culture and stimulated to undergo apoptosis with alendronate, apoptotic bodies recovered from the conditioned media contained RANK. These apoptotic vesicles were able to stimulate RANKL-reverse signaling and mineralization by osteoblasts as had been described by RANK-containing EVs^[24]^. The stimulation was blocked by the inclusion of recombinant soluble RANKL. Although this is an intriguing *in vitro* result, questions remain regarding whether this is a true physiologic mechanism. For example, when alendronate is given *in vivo,* it binds bone and reduces bone resorption by triggering osteoclasts that begin to resorb the treated bone to undergo apoptosis^[55]^. If apoptotic bodies were a significant anabolic signal, alendronate should be a potent bone anabolic agent. Instead, alendronate is able to reduce bone loss, but not stimulate increases in bone density^[55]^. It is possible that *in vivo*, surveilling macrophages detect and phagocytize apoptotic bodies from osteoclasts before RANK on the surface can stimulate RANKL reverse signaling in cells of the osteoblast lineage^[13]^.

### RANKL-containing EVs and cancer invasion

Numerous studies have suggested that cancer cells produce EVs that facilitate the growth and invasion of cancer cells to specific niches including bone^[56,57]^. Certain types of cancers, including breast, prostate, and multiple myeloma are particularly dangerous because of their ability to invade bone^[57]^. This is accomplished at least in part by sending signals that trigger inappropriate bone resorption by osteoclasts^[57]^. The “seed and soil” hypothesis proposes that that resorptive activity produces factors that trigger cancer growth and invasion and the stimulation of even more inappropriate bone resorption^[57]^. Might such cancer cells produce EVs containing RANKL to trigger bone resorption?

A recent study reports that EVs released by the breast cancer cell line MDA-MB-231 release EVs that contain the mRNA for RANKL^[58]^. However, RANKL was not detected by Western blot analysis, and cytofluorometric analysis also failed to convincingly demonstrate RANKL. These EVs were able to reach the bone microenvironment after intraperitoneal injection and be taken up by osteoclasts and osteoblasts. It was suggested that these EVs might be involved in deregulation of normal bone and endothelial physiology^[58]^.

### RANKL- and RANK- containing EVs outside of the skeletal system

As mentioned above, RANKL and RANK are found in tissues other than the skeletal system. There is some evidence that these cells may release and be regulated by EVs containing these proteins. One study identified RANKL and RANK in circulating EVs in humans^[43]^. They found that the level of RANK in the circulating EVs served as a biomarker for psoriatic, but not rheumatoid, arthritis. They also found that isolated circulating EVs could regulate bone cells *in vitro*. Subsequently, it was shown that circulating RANK- and RANKL- containing EVs serve as biomarkers for bone loss linked to antiviral treatment for HIV-infections^[44]^.

Might the RANKL in circulating EVs prove to be a better biomarker than overall circulating RANKL? Numerous studies have examined RANKL in circulation and in other body fluids as a possible biomarker for disease, with limited success^[59-65]^. These were performed under the assumption that circulating RANKL would all be the external domain cleaved by sheddases. However, we now know that some portion of these molecules is present in EVs. The absolute amount of these molecules in EVs, or ratios, like the amount of circulating cleaved RANKL divided by RANKL in EVs, may prove useful as biomarkers.

As described above, the RANKL/RANK systems are regulators in the brain^[23]^. For example, injection of RANKL into the brain causes thermoregulatory disruption in female mice^[21]^. EVs have been shown to pass the blood brain barrier, particularly under inflammatory conditions. It is possible that EVs in circulation that are derived from the brain can be isolated based on specific neuronal, or microglial markers, and then assayed for RANKL and RANK. These may prove useful biomarkers for neuroinflammatory or neurodegenerative diseases.

Finally, circulating RANKL has been shown to be a biomarker for cardiovascular disease^[63]^. Detection of circulating RANKL in EVs and perhaps circulating RANK in EVs, may improve the diagnostic performance.

## CONCLUSIONS

RANKL and RANK have been studied with great intensity since the discovery that RANKL was essential for stimulating osteoclast formation and bone resorption^[6]^. The therapeutic agent denosumab, a humanized monoclonal antibody that binds RANKL, has been successful for a decade for the treatment of osteoporosis and metastatic bone cancer^[8,9]^. RANKL and RANK have only recently been identified in EVs, and this area of study is still in its infancy. Already strong support for the hypothesis that RANKL and RANK-containing EVs have important roles in maintaining bone has emerged, as has evidence that these signaling molecules and their interactions can be targeted for therapeutics^[24]^ and used diagnostically^[42,44]^. The most exciting result so far has been the demonstration that certain crosslinked antibodies against RANKL can both inhibit bone resorption and stimulate bone formation^[24]^. Such bifunctional therapeutics might transform the treatment of bone diseases.

## DECLARATIONS

### Copyright

© The Author(s) 2021.
